# Sexual Dimorphism in Neurodegenerative Diseases and in Brain Ischemia

**DOI:** 10.3390/biom13010026

**Published:** 2022-12-22

**Authors:** Teresa Zalewska, Paulina Pawelec, Karolina Ziabska, Malgorzata Ziemka-Nalecz

**Affiliations:** NeuroRepair Department, Mossakowski Medical Research Institute, Polish Academy of Sciences, 5 A. Pawinskiego Str., 02-106 Warsaw, Poland

**Keywords:** sexual dimorphism, neurodegenerative diseases, Parkinson’s disease (PD), Alzheimer’s disease (AD), Huntington’s disease (HD), amyotrophic lateral sclerosis (ALS), multiple sclerosis (MS), brain ischemia

## Abstract

Epidemiological studies and clinical observations show evidence of sexual dimorphism in brain responses to several neurological conditions. It is suggested that sex-related differences between men and women may have profound effects on disease susceptibility, pathophysiology, and progression. Sexual differences of the brain are achieved through the complex interplay of several factors contributing to this phenomenon, such as sex hormones, as well as genetic and epigenetic differences. Despite recent advances, the precise link between these factors and brain disorders is incompletely understood. This review aims to briefly outline the most relevant aspects that differ between men and women in ischemia and neurodegenerative disorders (AD, PD, HD, ALS, and SM). Recognition of disparities between both sexes could aid the development of individual approaches to ameliorate or slow the progression of intractable disorders.

## 1. Introduction

**Figure 1:**
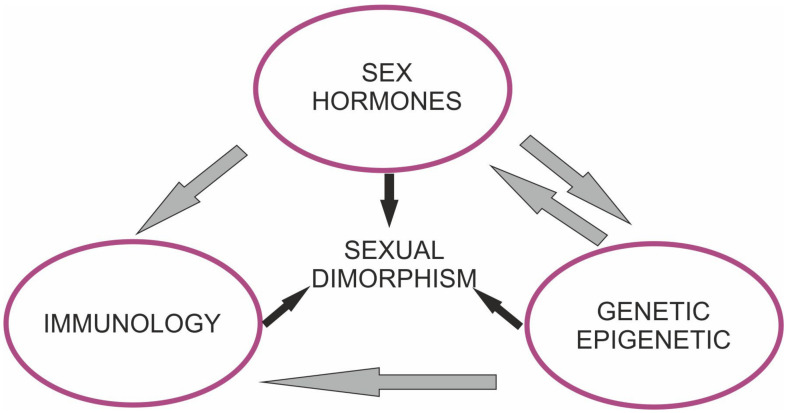
Mechanisms regulating sex specific responses to neurodegenerative diseases and cerebral ischemia.

Brain dimorphism is a complex process, with multiple contributing mechanisms and pathways resulting in differences. Sex-based differences with regard to clinical features have been identified in a range of neurological diseases, including Alzheimer’s disease (AD), Parkinson’s disease (PD), Huntington’s disease (HD), amyotrophic lateral sclerosis (ALS), multiple sclerosis (MS), and ischemic injury [1,2,3,4,5,6]. The difference in response to brain pathology plays a role in the prevalence and natural course of several disorders. The responsible mechanism of dimorphism may vary in species and involve several factors, including epigenetic and genetic differences, hormonal effects, sex-specific environmental factors, and gene–environment interactions (Figure 1). A strong connection between sex hormones and the development of sex differences in the brain has been noted. However, other unidentified factors may also play an important role [7]. Despite recent advances, the precise link between the considered factors and brain disorders is incompletely understood. We provide up-to-date data on sex-related differences in ischemia and neurodegenerative disorders and highlight the most relevant aspects that differ between men and women. Increased recognition of such differences may facilitate the introduction of potential personalized therapy to ameliorate intractable disorders.

## 2. Neurodegenerative Diseases

Neurodegenerative diseases constitute a heterogeneous group of disorders that increase in incidence as the population ages. These disorders are characterized by a progressive decline in cognitive ability and memory formation, which are correlated with reduced neurogenesis and deficits in LTP maintenance in elderly people [8]. Increasing brain and spinal cord damage gradually impairs the function of the central and peripheral nervous systems. This increasing damage finally leads to mental retardation and motor and behavioral problems. The most common neurodegenerative diseases include Parkinson’s disease (PD), Alzheimer’s disease (AD), Huntington’s disease (HD), amyotrophic lateral sclerosis (ALS), and multiple sclerosis (MS). Detailed systematic studies of brain pathology have detected the role of sex-dependent effects in the course of diseases. Since then, new research has emerged, providing an unprecedented view into the differences between the sexes related to the severity of disease symptoms, progression, and different treatment responses. In the following pages, we present the current data on this subject.

### 2.1. Parkinson’s Disease

Parkinson’s disease (PD) is a common age-related neurodegenerative disorder of the CNS, affecting 1–2% of the population over the age of 65 [9]. The main features of this pathology are progressive motor dysfunction, such as hypokinesia, resting tremors, rigidity, and postural instability. Moreover, nonmotor symptoms, such as olfactory deficits, constipation, sleep behavior disorders, mood disturbances, and dementia are also observed in PD patients [10,11]. The neuropathological hallmarks of PD are intracellular protein aggregates called Lewy bodies, in which α-synuclein is the principal component, and the degenerative processes of dopaminergic neurons in the substantia nigra pars compacta (SNpc), which cause depletion of dopamine in striatal projections [12,13]. Although the mechanism that triggers brain degeneration in PD is unknown, several genetic and epigenetic factors are involved in contributing to the disease. A comparative clinical and experimental study showed that males have a greater susceptibility to PD than females, with a 1.5–2.0-fold higher prevalence [14,15]. The differences between the two sexes are related to the clinical course of the disease, severity of symptoms, progression, and treatment responses [1,16,17]. For instance, men need higher doses of L-dopa than women to achieve optimal therapeutic control [18].

Another set of supporting information came from studies of animal models, which recapitulated the striatal dopaminergic neuron loss by administration of toxins (MPTP, methamphetamine, 6OHDA). The results have revealed the higher susceptibility of males (rodents and nonhuman primates) in vivo and in vitro [19,20]. Of note, sex differences have been detected only when partial lesions of the nigrostriatal dopaminergic pathway do not exceed 70–80% damage [21,22]. Finally, the majority of data implied that sex is an important factor in PD development.

**Figure 2:**
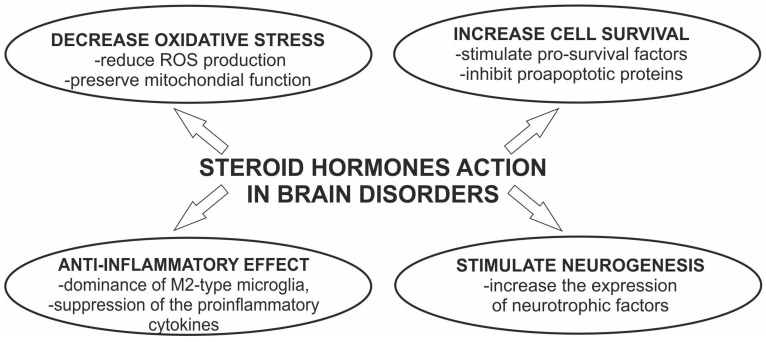
Neuroprotective effects of steroid hormones in brain disorders.

Further systemic studies in the field of PD showed that sex hormones played a neuroprotective role in multiple aspects of PD as well as in other brain disorders (Figure 2). Administration of hormones to postmenopausal women reduced the risk for disease, as was already noted in other neurodegenerative impairments [23,24,25]. Thus, the action of estragon and 17β-estradiol (E2) have received considerable attention, spurring justified hope as therapeutic strategies against the development of PD [1,22].

One of the most important actions of estrogens is related to a defense of neurons in the nigrostriatal dopaminergic pathway from potentially harmful toxic stimuli [26]. Furthermore, estrogen is postulated to prevent Levy body formation and aggregation of α-synuclein [27]. One further notable advance is the recognition that sex hormones, in addition to direct action on DA neurons, may also influence input circuitry in a sex-dependent manner. To date, evidence has shown that circulating estradiol upregulates the expression of TH (the rate-limiting enzyme for noradrenaline as well as DA synthesis) in females. In males, circulating testosterone downregulates TH after its conversion to estradiol [28].

The effective protection of dopaminergic neurons by estrogen is mediated by both nongenomic actions, such as the activation of specific signaling pathways, and genomic effects involving gene transcription. For excellent reviews, please refer to Jurado-Coronel et al. and Gillies et al. [1,29]. In brief, the model of actions is associated with decreasing oxidative stress, reduced production of ROS, stabilization, and preservation of mitochondrial function, and anti-inflammatory effects [30,31]. The detailed analysis of the intracellular pathway associated with 17β-estradiol showed that stimulation of the MAPK/ERK and PI3/AKT cascades is linked to cell survival by inhibition of proapoptotic proteins. In addition, the possible linkage of the neuroprotective 17β-estradiol with the expression level of neurotrophic factor GDNF in the SN and striatum has also been postulated [32]. Although the entire spectrum of molecular events resulting from hormone treatment has been described, to date, there is no information on whether the intracellular signaling pathway is sexually differentiated due to the differential expression of molecules associated with the cell survival pathways.

Despite these promising results regarding the neuroprotective effect of in neurotoxic animal models [33,34,35], to speculate that hormones would be able to ameliorate pathological reactions in human PD would be rather premature. Indeed, some clinical studies have reported no evidence of the neuroprotective effects of estrogen [36,37]. In addition, the disparity in reported findings does not allow us to make precise conclusions [26,35]. Therefore, more work is needed to fully decipher the mechanism of hormonal sex action in PD patients.

In the line of gathering data, sex hormones alone do not entirely explain sex differences. Lee et al. [38] highlighted the important role of sex chromosome genes. According to Gillies et al. [1], SRY, which is present only in males, contributes to sex differences. Of note, it might directly influence the nigrostriatal dopaminergic system. Moreover, it was upregulated in an animal model of PD [39]. Importantly, silencing of the SRY gene in the SNc of male rodents reduced the DA neuron number compared with that of females and induced motor deficits [1]. These data suggest that lowering nigral SRY expression would represent an important event in future sex-specific strategies to slow or prevent DA cell loss in PD males [38]. Another important aspect of genetic studies performed in PD has shown that genetic variations in ERβ are associated with an early age (between 20 and 50 years) of PD onset [40,41,42].

In summary, the common interplay of chromosomal factors and gonadal hormone factors may contribute to sex differences.

### 2.2. Alzheimer’s Disease

Alzheimer’s disease (AD) is one of the most common neurodegenerative disorders and the leading cause of death for individuals aged 65 or older. AD accounts for approximately 80% of all cases of dementia, due to progressive cognitive impairment and decreased memory formation associated with neuronal dysfunction [8]. The two pathological hallmarks of AD are extracellular senile neuritic plaques, of which amyloid-β is the principal component [43], and intraneuronal accumulation of hyperphosphorylated microtubules associated with the protein tau, which are known as neurofibrillary tangles. The aggregation of amyloid-β fragments (peptides 40–42) that accumulate to form oligomers induces neurotoxic effects that lead to the neural synaptic and cognitive degradation seen in AD [44]. Intracellular neurofibrillary tangles, as the second hallmark of AD pathology, are involved in the dispersion of microtubules and contribute to the progression of the disease [45].

Clinical data has shown that women have a greater risk of developing AD than men. The datasets present a higher incidence of Alzheimer’s disease, at a ratio of approximately two to one [46,47], and women experience more progressive cognitive and physical decline [48,49,50,51]. It must be also noted that although women have a longer lifespan, they showed a significantly faster decline and greater deterioration of cognition than elderly males.

Evidence of a higher prevalence and higher level of AD pathology in women was found by a number of detailed postmortem analyses of brain AD patients [52]. This investigation revealed a heightened tau tangle density, higher amyloid-β load, and greater magnitude of brain atrophy in women than in men. In addition, positron emission tomography (PET) was used to detect amyloid-β load and tau deposits, which appeared earlier in women than in men in individuals at risk of developing AD [53]. Furthermore, sex differences were also identified in an animal model of triple transgenic mice (3xTg-AD mice) [54]. It is worth pointing out that other transgenic mouse models have been also used to study the cognitive and behavioral deficits associated with AD pathology. These include APP/PS1 [55,56,57] 5xFAD, Tg2576 [58,59], and APP23 mice [60]. Therefore, based on the above findings, deposits of amyloid-β were more striking in females than in age-matched males. This observation corroborates previously published reports showing more senile plaques [61,62].

Clinical studies strongly argue in favor of a prominent role of the apolipoprotein E (epsilon-4 allele (APOE4) being a greater risk for developing AD in women in contrast to age-matched men [63,64,65]. The research data indicate that the risk of AD is even more pronounced in women who are carriers of the e4 allele of APOEthan it is in e-4 carrying men [64,65]. In contrast, a limited number of studies did not find an interaction between sex and APOE epsilon-4 [52,66]. According to this data, women with the APOE genotype do not present a more severe phenotype than men, and higher pathological changes were thus not related to the APOE status. However, this is yet to be determined in future investigations.

Accumulating evidence shows that the age-related drastic loss of sex hormones, resulting in the depletion of estrogen post-menopause, leads to the higher prevalence of AD in women [67,68]. Evidence from animal models has shown the neuroprotection of steroid hormones in AD [69]. An important point for supporting this suggestion is that ovariectomized mice present early and accelerated AD pathology and impaired learning, while 17β-estradiol prevents the accelerated decline [70,71]. Importantly, estradiol decreases amyloid-β levels and its aggregation into plaques in female mice expressing APP compared with wild-type mice [72]. A limited number of studies have aimed to decipher the molecular pathway underlying beneficial estrogen action. One of the postulated mechanisms is linked to the enhancement of α secretase activity and consequently directs APP processing towards a nonamyloidogenic pathway [73,74]. The other mechanism involved a reduction in amyloid-β levels through increased inhibition of BACE1 and increased amyloid-β clearance [75]. Notably, the protective action of hormones has also been shown in other systems. There are data showing that estradiol induced dephosphorylation of tau and protected against tau hyperphosphorylation in female neuroblastoma cell lines and cortical neurons [76].

There is some evidence that a high serum level of follicle-stimulating hormone (FSH) is strongly associated with the onset of AD. FSH acts directly on hippocampal and cortical neurons to accelerate amyloid-β and Tau deposition and impair cognition in 3xTg AD mice. Blocking FSH action in these mice abrogates the Alzheimer’s disease-like phenotype by inhibiting the neuronal C/EBPβ–δ-secretase pathway [77]

Although the reported preclinical data seem to suggest a protective role for estrogen in AD pathogenesis, clinical trials have resulted in inconclusive data, with some showing decreased cognitive function with early estrogen therapies [78,79,80,81,82]. In addition, long-term therapy resulted in negative effects and increased the risk for breast cancer, pulmonary embolism, and stroke.

In men, the loss of steroid hormones is less drastic. The level of circulating testosterone, the main male sex hormone, presents a gradual reduction with time, but its decreased levels with ageing may also increase AD in men. This is supported by gonadectomized males showing increased amyloid-β accumulation and memory-related behavioral deficits, which were attenuated by treatment with testosterone or its metabolite dihydrosterone [83,84,85]. Another study indicated that increased testosterone in aged male 3xTg-AD mice was correlated with reduced amyloid-β plaque pathology [86].

Apart from hormones, genes are likely to explain more AD pathology in women. As a result, ubiquitin-specific peptidase 9 has highly significantly different expression in women compared with men and is associated with the expression and phosphorylation of tau [87]. In addition, three genes were identified in a sex-stratified genome-wide association study that was associated with tau (osteocrine and claudin-16) and amyloid-β (serpin family B member 1) only in the brain of women [88]. Furthermore, several other genes have been identified recently such as Kdm6a [89], MGMT variants [90], ubiquitin-specific peptidase 11 [91], and St2 [92]. A full understanding of the role these genes in AD requires further study.

A body of evidence implies that the differences in immune system activation may be a causative factor in AD differences, as females present a stronger immune response than men with increased inflammatory cytokines, chemokines and gliosis [51,93,94]. This may include changes in gene transcription in microglia. However, the mechanism of action remains unclear. In addition to the effect discussed above, several molecular pathways, associated primarily with sex hormones reduction, may play a role in the determination of female AD vulnerability. The study clearly demonstrates that oxidative stress is one of the major pathogenic factors of AD, which is closely associated with other key events such as a decline in mitochondrial function, protein misfolding, widespread neuronal/synaptic dysfunction, altered neuronal ionic homeostasis, impaired kinase/phosphatase activities, selective neuronal loss with attendant neurotransmitter deficits and impaired autophagy [95,96]. Future studies will need to address the complexity to achieve an understanding of the complex mechanism.

### 2.3. Multiple Sclerosis

Multiple sclerosis (MS) is a chronic autoimmune demyelinating disease of the CNS with a variable pathology and phenotypic presentation. MS has many characteristics in common with classical neurodegenerative diseases. The disease is characterized by recurrent episodes of inflammatory demyelination with a relapsing-remitting course, significant synapse loss, and CNS atrophy [97,98,99]. Due to epidemiological data, MS is more prevalent in women than in men, with a ratio of 3:1 [100], and mainly affects young and post-pubertal women. Interestingly, an alleviation of disease activity observed during the late state of pregnancy might be associated with suppressing the mother immune system to prevent rejection of the fetus [5,101,102].

Sex-related differences between males and females, seen in humans and animal models, are characterized by the incidence of disease, its activity, and its progression. Subsequent research discovered that the disease onset, disease severity, and faster accumulation of disability appear to be greater in males, although they have a lower risk of developing MS than females [5,100,103,104,105,106]. Other datasets indicate that the neurodegenerative component is most pronounced in male patients, whereas females present higher inflammatory activity [107]. Male mice bearing the XY genetic background demonstrated greater EAE (MS model) disease severity and more pronounced neurodegeneration than mice with XX chromosomes. In summary, sex-related factors may be responsible for a higher susceptibility to immunity in females vs. males, and may play a crucial role in the etiopathogenesis of MS [3,108]. The notable advance is the clinical findings consistent with observation that male sex confers an increased risk for worse disease progression [109]. The voxel-based morphometry analysis, showed worse grey matter atrophy and cortical thinning in MS men but not in MS women. It was associated with worse upper extremity of function, as shown by significantly worse performance. It is concluded that grey matter atrophy is a sensitive putative biomarker for clinical disability progression. Considerable attention has been particularly directed, as in other neurological diseases, to the role of sex hormones and their effects on immune function.

Increasing clinical data show that sex hormones (estrogen and testosterone) are therapeutically effective in animal models of MS. A detailed study indicated that both steroids derived from the peripheral system and neurosteroids (steroids synthesized in neural cells) likely exert variable and complex inflammatory and neuroprotective effects in the course of MS. Notable advances have shown that steroids are not only involved in the immunological response in MS but also interact with intrinsic brain cell species. The specific action on CNS cells, like in the peripheral system, is principally mediated by respective receptors (ERα, -β, and G-protein coupled receptor 1), which coordinate multiple signaling mechanisms that may protect the brain from neurodegenerative factors [110]. For instance, estrogen receptor α signaling in peripheral immune cells has been shown to be the main pathway for the protective effect of estrogen in experimental EAE [111].

CNS inflammation and axonal loss are closely connected with the expression of the receptor ERα in astrocytes but not neurons [112]. Subsequent research showed that the ERβ receptor present in oligodendrocytes enhanced endogenous remyelination and increased mature oligodendrocytes in animal models (EAE and cuprizone models) [113,114,115]. More recently, a direct effect on the immune system mediated by ERβ stimulation has also been described [111]. Selective ERβ modifiers transcriptionally inhibit the propagation of inflammatory signals in microglia [116,117]. Notably, estrogen signaling, in addition to classical receptors, contributes to protective immunomodulatory effects mediated by G-protein coupled receptor 30 (GPR30) [118].

Further systematic studies showed that not only estrogen but also other hormones (testosterone, progesterone, and allopregnanolone) can potentially exert beneficial therapeutic potential in MS. Important results in these datasets are the finding that testosterone and its synthetic analogue may result in increased myelin formation and promote repopulation of oligodendrocytes [119,120] in EAE. Likewise, progesterone promotes myelin repair and reduces neuroinflammation [121]. As noted in other reports, progesterone and its analogue also improve clinical scores and decrease neuronal pathology [119,122].

There are no detailed data on the downstream signals stimulated by hormones in MS; however, the response outside of MS was described by Ramien et al. [3]. Nevertheless, it is obvious that the downstream signaling pathway may be specific to the disease model of neurodegeneration. Therefore, it cannot be fully accepted as a specific effect on MS.

Despite the established fact that numerous in vivo and in vitro studies provide growing evidence that sex hormones show promising neuroprotective effects in MS, they may play even opposing roles in the course of disease. One example is testosterone. Although it ameliorated the immune response in the early stage of EAE, it was detrimental to neurons during the chronic phase, with neurodegeneration being more pronounced in male patients [108,123]. Similar biphasic action presents endogenous ER agonist 17β-estradiol. This factor demonstrates anti-inflammatory action associated with higher physiological levels of estrogen, while lower levels promote the production of proinflammatory mediators. Interestingly, clinical phase trials have been conducted for testosterone in male patients and estrogens in female patients with MS [108]. The authors report a significant effect on a measure of cognition and during a treatment case: slowing global brain atrophy and altered immunological profiles [124]. However, until now, there has been insufficient knowledge about the role of sex with regard to treatment response. The treatment for MS may require different therapeutic approaches in males and females due to disparities in the course of disease.

Currently, we know that sex hormones exert complex and indirect effects on immune function, which likely contributes to the sex bias in the incidence of autoimmune diseases in the CNS. Sex hormones are likely implicated in complex interactions with environmental (sunlight and vitamin D) genetic and epigenetic factors to influence MS risk and progression. They can also indirectly affect immune function by altering the composition of the microbiome, as sex-specific differences in the microbiome are present in several mouse strains and affected by hormone treatment [125]. In addition, some data suggest that sex differences in immune responses may also be influenced by direct genetic effects independent of sex hormones [126]. Future studies investigating the potential role of the factors driving disease disparities between males and females are needed.

### 2.4. Amyotrophic Lateral Sclerosis

Amyotrophic lateral sclerosis (ALS) is a fatal neurodegenerative disease associated with progressive degeneration of upper and lower motor neurons. Damage to motor neurons and denervation of neuromuscular synapses in the peripheral nervous system result in the loss of control of voluntary muscle movements, spasticity respiratory failure, and ultimately paralysis and death within 2–5 years of diagnosis [127,128]. Although the exact mechanism precipitating motor neuronal death is not precisely defined, subsequent analyses of animal models and human patients identified a plethora of pathological events in the course of ALS, including synaptic terminal degeneration, glial cell activation and sustained upregulated immune responses, accompanying the pathology of ALS [129,130,131]. However, the primary process leading to ALS pathology remains a matter of discussion.

Current evidence found that some aspects of ALS onset and progression are dependent on sex in patient and animal models of the disease. The results of an epidemiological study indicated that women are less susceptible to developing ALS and exhibit less severe disease progression [79,132,133,134,135]. Furthermore, females usually live longer than males, despite having a similar symptomatic stage duration. The general data from humans are mirrored in a mouse model of ALS and confirmed that the development and progression of disease are sex-dependent [136,137]. One notable advance of a subsequent study was the observation that sex-dependent differences became less significant as patients aged. After data-based inspection showing the age-dependent course of disease between men and women and the fact that postmenopausal women are just as likely to develop ALS as men, it was assumed that sex hormones contribute significantly to the regulation of these processes [138].

A further study suggested a protective effect of female sex hormones, primarily estrogen and progesterone, in the course of disease. For instance, exposure to endogenous estrogen significantly increases survival time in postmenopausal women with ALS [139]. In addition, supplementation of ovariectomized females with a high dose of 17βestradiol (E2) resulted in an extended lifespan [140,141]. Subsequently, deficiency of endogenous estrogen has been demonstrated to have a deleterious effect in female transgenic SOD1 mice exacerbated by acceleration of the disease, making disease progression and lifespan comparable with those of male mice [140,141,142]. Understanding the role of sex hormones in ALS has proven more difficult. Unfortunately, limited research has examined the effect of exogenous hormones on disease onset and progression in females. The analysis of a small cohort of ALS patients yielded contrasting results [143,144]. However, despite several inconsistencies, the results indicate the involvement of sex hormones in ALS [145].

Surprisingly, van der Berg et al. [146] and Murdock et al. [147] described sex as an important variable in the most recent ALS clinical trial. Sex-specific factors in the immune system were hypothesized to contribute to the observed pathological and clinical differences between males and females in neurodegenerative diseases [148]. Considering the recent interest in ALS immune factors and the increased number of clinical trials targeting the immune system, this observation appeared to be important. It is also known that autoimmune diseases, such as systemic lupus erythematosus and MS, disproportionately affect females [94].

Growing data indicate that sex differences may also be influenced by direct genetic effects. The recent report of Santiago et al. [149] identified a set of switch genes exhibiting sex-specific gene expression patterns in the blood of ALS patients. The detailed functional analyses found that disruption of male switch genes of subsequent biological pathways in ALS was associated with alteration of metabolic and energetic pathways. In contrast, pathways related to infection, inflammation, and apoptosis were more prominent in females. Future studies aimed at determining the role of these genes in sex differences are needed.

Finally, based on the important role of miRNAs and their abnormal expression in various muscle disorders, only a few studies have focused on their action in ALS muscles [150]. One piece of research focused on the investigation of miRNA-206 expression, the key regulator of signaling between muscle fibers and neurons [151,152,153]. Despite the differential miRNA expression in ALS muscle from male patients compared to females, further studies provided discordant results.

In summary, despite key findings, the sex-dependent mechanism remains unclear, and much uncertainty remains regarding the roles of other factors.

### 2.5. Huntington’s Disease

Huntington’s disease (HD) is an autosomal dominant neurodegenerative disease (recently reported peripheral tissue involvement) caused by genetic mutation in the huntingtin gene (HTT) that leads to tandem CAG repeats. The expansion of CAG repeats is recognized as the main risk factor for developing HD and presenting clinical symptoms, including a variable combination of movement disorders, cognitive impairment and behavioral symptoms, progressing to worsening function [154,155]. Notably, in addition to CAG repeat length, other genetic factors may contribute to HD phenotype expression and determine the age at onset of HD. For instance, there is evidence demonstrating that the apolipoprotein E epsilon 2/epsilon3 genotype is associated with an earlier age of HD onset in male than female patients [156,157]. In addition, a coding variant in PPARGCIA is associated with earlier motor onset in male HD gene carriers [158]. The described divergent role of these genetic factors emphasizes sex differences, which were, in contrast to other neurodegenerative diseases, almost not highlighted in HD. Scientists have largely thought that HD is sex independent due to an autosomal inheritance pattern [159]. A sex bias emerged from epidemiological analysis of HD patients as well as from HD animal models only in past decades [51,160,161,162,163,164]. Since then, a significantly higher prevalence of HD in women has been detected [165]. In addition, women suffer a more severe disease phenotype and faster progression, particularly in the motor and functional domains, than men [166]. Motor symptoms have a stronger impact on functional ability in women than in men with HD [159]. Notably, other studies did not disclose any sex-related effect of HD progression or clinical phenotype [167]. Finally, in contrast to human studies, the results of animal model investigation indicate a more severe picture in males. Males exhibited stronger motor deficits and more prominent neuropathology.

The field of HD research has recently focused on the relative contribution of sex to depression. Depression is part of early onset and the most common effective symptom in Huntington’s disease [168,169]. Clinical data showed a higher prevalence of depression in women than in men [159,170], although symptom progression over the years was similar between the sexes. Nevertheless, female sex was postulated to be a predictor of increased severity of symptoms [171]. In contrast, other experimental evidence postulated that disease stage, rather than sex, is related to depressive symptoms in HD [172,173]. This is consistent with the observation of no connection between sex differences and depression in other neurodegenerative conditions, such as PD [174] and MS [175].

Surprisingly, the next series of experiments found sexually dimorphic depressive-like behaviors at a premotor symptomatic age in R6/1 transgenic mice but only in a female mouse model of HD [176,177,178]. The increased rate of depression in female patients might be associated with the reduction of testosterone in animal serum [162,179] as well as in HD patients [180,181,182]. In some studies, supplementation with E2 had a beneficial effect on postmenopausal depression [183,184]. However, data showing a proper correlation between clinical symptoms and lifetime estrogen exposure in women with HD are limited due to the young age of onset of the disease. Nevertheless, the protective effect of sex hormones in females on neurodegeneration related to HD progression was further reported by several authors [161,162,164], but the results are inconsistent and incomplete. The published controversies did not allow us to make conclusions. Further investigation is necessary.

## 3. Sexual Dimorphism following Brain Ischemia

Stroke is defined as a clinical syndrome characterized by the rapid onset of focal (or global, in the case of subarachnoid hemorrhage) cerebral deficit lasting more than 24 h or leading to death due to a vascular cause [185]. Among the different stroke subtypes, approximately 80% of strokes occur following occlusion of the cerebral artery. Stroke is a leading cause of morbidity and mortality and a major cause of public health burden worldwide. Human clinical and epidemiological data indicate that ischemic injury is sexually dimorphic. Elucidating the origins, mechanism and impact of sexual differentiation of the ischemic brain has been a topic of investigation for many years, and many important principles have been established. Males have been found to have a higher incidence of stroke and poorer outcome afterwards [186]. Additionally, numerous in vivo experimental studies in models of forebrain or focal ischemia provide growing evidence that young adult female rodents sustain smaller infarct sizes than males in the same types of stroke [187,188,189,190]. However, with advancing age, the incidence of stroke becomes higher in women and is correlated with a decline in estrogen levels after menopause, which puts females at a higher risk [191,192,193]. To further address the beneficial role of estrogen, treatment with this hormone was found to reduce brain damage after MCAO. Therefore, the noticeable effect of sex and age on the incidence and outcomes from ischemic stroke points to sex hormones as factors that determine sex-specific responses to the injury [194].

Ischemic injury initiates a number of pathological processes that are each responsible for brain damage. There is full agreement that a critical role in the pathophysiology of ischemia-induced brain damage is neuroinflammation driven primarily by activated microglial cells [195,196]. The specific contribution of microglia to brain inflammation as well as soluble mediators of the inflammatory response to ischemia continue to be a focus of many valuable publications [197,198,199].

Upon activation by ischemic stress, microglia become polarized and exhibit different expression patterns and morphology in males compared to females and demonstrate a specific immunological response [200]. Thus, the concept of sexual dimorphism came from recent advances that classified the functional and morphological phenotypes of microglia and provided new insight into sexual dimorphism after ischemia. Since then, specific microglial phenotypes have been believed to have a significant impact on the evolution of ischemic injury.

The microglial phenotypes were classified as either proinflammatory M1 (the so-called “sick”) or M2 (called “healthy”) [201], however, transcriptome studies have shown that in vivo microglia activation is varied, meaning that M1 and M2 represent a spectrum of activation patterns rather than separate cell subtypes [202], and there is a continuum of different intermediate phenotypes in microglia [203]. Although the binary concept of microglial M1/M2 classification has recently been debated, classifying microglia function as either neurotoxic (M1) or neuroprotective (M2) is useful for explaining the pathobiology of inflammatory and degenerative CNS disorders [204,205]. After ischemic injury, M1 microglia produce proinflammatory mediators and neurotoxic molecules (cytokines IL-1β, TNFα, chemokines, ROS, and NO). In contrast to M1 action, M2 displays both immunosuppressive and neuroprotective properties due to the release of anti-inflammatory as well as neurotrophic factors (IGF, transforming growth factor (TGFβ), GDNF, IL-4, and IL-10) that promote brain repair and regeneration [206]. Therefore, both microglial phenotypes play a role in the inflammatory response after CNS injury, but the extent to which each phenotype is involved is not yet fully understood.

When comparing the two sexes, the number of M2 phenotypes (determined by expressing polarization markers Ym1 or Arg1) was higher in female individuals and most likely contributed to the lower risk for ischemia [207]. The finding that microglia from female mice possess neuroprotective ability was next confirmed by Villa et al. [198]. They found that the M2 phenotype retains this functional ability when transferred into the brains of male mice.

Another set of informative data came from analyzing the effect of ischemia on mitochondria. The findings from these studies implied that mitochondria are a key point of sexual dimorphism [100,208]. Detailed investigation of the mitochondrial response to ischemia showed that males are more sensitive to oxidative stress with greater production of ROS, increased mitochondrial permeability and greater release of mitochondrial proteins. Surprisingly, females present increased activity in antioxidant enzymes [209]. However, there is still insufficient information regarding the mitochondrial contribution, and this subject requires further investigation.

Finally, emerging definitive evidence from genomic data show that candidate genes and short coding microRNAs regulate gene expression and are also involved in the pathophysiology of ischemia/reperfusion injury. Investigation of epigenetic regulatory mechanisms and posttranslational modulation of gene expression has gained increasing attention and led to the suggestion that these processes may account, at least partially, for differences between sexes [210,211,212,213].

A few reports have addressed the role of short noncoding microRNAs. Indeed, the investigation showed differential expression of poststroke circulating miRNAs between male and female rats, suggesting that miRNAs modulate sex-dependent changes in cerebral ischemia [214]. However, only miR-375 has been examined in an in vivo stroke model, and only in male animals. However, the augmentation of estrogen signaling pathways associated with miR-375 treatment suggests that this miRNA may play a contributory role in sexually dichotomous outcomes following stroke [215].

An elegant investigation has revealed that sex-specific differences characterize cellular signaling and death pathways [216]. Evidence from various studies points subsequently to cell death, dependent mainly on caspase activation in females, while the polyADP polymerase-1 (PARP) pathway predominates in males [217]. Different cell death pathways after hypoxia-ischemia determine the differences in sex-dependent responses to pharmacological intervention. For instance, treatment with 2-imminobiotin as well as a caspase inhibitor provided neuroprotection in females only [218,219,220]. In contrast, nitric oxide inhalation improved the outcome in males only [221]. Moreover, minocycline, a drug that works in part as a PARP inhibitor, was found to be effective in protecting male animals after stroke [222]. Considering these data, individual therapy is needed.

An increasing body of evidence emphasizes the importance of estrogen as a neuroprotective agent. Surprisingly, only a few studies have assessed whether sex-specific signaling pathways are hormone dependent. Preclinical estrogen or β-estradiol acts as a neuroprotective factor in experimental stroke by reducing inflammation and oxidative stress and sustaining neurogenesis [223,224,225,226]. Females have been found to be more protected from brain injury pre-menopause, with high levels of circulating estrogen. Recent evidence suggests that chronic estrogen deficiency in postmenopausal women allows increased activation of immune-related genes [227] and thus an exaggerated response to ischemic injury, while an increased risk of stroke is noted when estrogen levels decrease. However, clinical studies of hormone replacement have potential harmful effects in humans, including breast and endometrial cancers as well as thrombosis [227]. The relationship between sex hormone exposure and ischemic stroke risk still appears complex. The use of hormones requires a careful timing dosage, and the age of the recipient should be considered, as it could modify the effect of sex hormones on stroke outcomes [119,194,210,228,229]. In conclusion, hormonal manipulation alone has proven to be ineffective in improving stroke outcomes in human trials, reflecting the sexually dichotomous cell death and regeneration pathways induced following cerebral ischemia.

## 4. Conclusions

The presented data showed sex differences in response to neurodegenerative diseases and brain ischemia. The disparities between males and females are commonly observed in patients and pre-clinical models. Through complex correlations and interactions of several factors, this phenomenon influences the natural course of the diseases by influencing multiple facets, including pathogenesis, clinical features, and overall management. However, the precise link between contributing factors and brain disorders is not clear. Elucidating sex differences in disease phenotypes will be very instrumental in the development of sex-specific strategies for prevention, detection, and treatment. The methods are constantly improving with the potential to identify factors driving disease differences between males and females. According to this review, much research needs to be done in the near future.

## Data Availability

Not applicable.
